# Draft genome sequence of *Desulfobacterota* strain M19, a mesophilic sulfur-disproportionating bacterium from a deep-sea hydrothermal vent on the Mid-Atlantic Ridge

**DOI:** 10.1128/mra.00295-25

**Published:** 2025-06-09

**Authors:** Marie Hemon, Lukáš Novák, Maxime Allioux, Léna Ailliot, Erwann Vince, Karine Alain

**Affiliations:** 1 Unité Biologie et Ecologie des Ecosystèmes marins Profonds BEEP, IRP 1211 MicrobSea, EMR 6002 BIOMEX, CNRS, Ifremer, Univ Brest 668293, Plouzané, France; 2 Research Center for Bioscience and Nanoscience (CeBN), Research Institute for Marine Resources Utilization, Japan Agency for Marine-Earth Science and Technology (JAMSTEC) 13570, Yokosuka, Kanagawa, Japan; DOE Joint Genome Institute, Berkeley, California, USA

**Keywords:** sulfur disproportionation, hydrothermal vent, sulfur oxidation, autotrophy

## Abstract

The draft genome sequence of *Desulfobacterota* strain M19, likely representing a new genus, is reported. It was isolated from a deep-sea hydrothermal vent and is capable of autotrophic growth by anaerobic disproportionation of elemental sulfur. Its genome has been sequenced because there are few publicly available genomes of sulfur-disproportionators.

## ANNOUNCEMENT

Anaerobic sulfur disproportionation is a singular metabolic pathway where a sole intermediate sulfur species is reduced and oxidized to produce energy. The genetic basis for this metabolism is still only partially deciphered.

Strain M19 (UBOCC-M-3423^T^) was isolated from a hydrothermal rock sample (Capelinhos site, Lucky Strike vent field, Mid-Atlantic Ridge) collected using the *Nautile* submersible’s clamp, placed in an insulated box (Sample MOM19 Cap2 PL1945-7; N 37° 17.358’ W 32° 15.834; depth: 1663 m; 19 June 2019), and stored at 4°C under N_2_ (1). Isolation was performed at 19°C, by serial dilutions to extinction, in a mineral basis with 5 g.L^–1^ S^0^, 34.5 mM Fe(OH)_3_, and CO_2_ (100%) (2). Strain purity was confirmed by genome and 16S rRNA gene sequencing. M19 grows chemolithoautotrophically by S^0^ disproportionation, producing sulfide and sulfate (detected by colorimetry and ionic chromatography).

Genomic DNA was extracted using a phenol-chloroform method (3). Short-read and long-read DNA sequencing were conducted using the Illumina nano-MiSeq (2 × 150 bp paired-reads, Nano V2 chemistry kit; 550,146 reads) and the Oxford Nanopore MinION technologies (R9 Flow Cell FLO-MIN106, Rapid Sequencing kit SQK-RAD004; 16.11 Mb; N50 = 7.95 kb). Quality controls were performed by sequencing facilities and with FastQC v.0.12.0 (4). Reads were assembled using Unicycler (v0.4.9) (5). Genomic annotation was performed using the MaGe platform (v3.17.3) (6). Pairwise 16S rRNA sequence similarity was calculated using the EzTaxon-e server (7) and average amino acid identity (AAI) scores using EzAAI (v1.2.3) (8). Default parameters were used for all software. Genes of the sulfur cycle were searched using custom HMM profiles with the hmmsearch tool of HMMER (v3.4) (9).

The draft genome sequence consisted of 90 contigs, with a size of 3,092,747 bp, a G + C content of 49.35%, an N50 of 261,071 bp, and a genome coverage of 51.67×. MaGe annotation predicted 2,882 CDS, including 45 tRNA genes and one 5S-16S-23S rRNA operon. CheckM (v2) (10) estimated the genome to be 97.62% complete, with 1.19% redundancy.

GTDB-Tk (v2.4.0+) (11) assigned strain M19 to the *Desulfobulbales*. Its closest relatives were *Desulfotalea arctica* LSv514^T^ (90.26% 16S rRNA gene similarity), *Desulfopila inferna* JS_SRB250Lac^T^ (89.79%), and *Desulfosediminicola flagellatus* IMCC35005^T^ (89.79%). AAI scores were 57.30% for *Desulfosediminicola flagellatus* and 57.20% for *Desulfopila inferna*. These values fall below genus-level thresholds (<94.5% 16S rRNA gene identity [12]; <65% AAI [13]), suggesting that strain M19 likely represents a new genus.

A complete dissimilatory sulfate reduction pathway is encoded in the genome (Sat, AprA, AprB, DsrA, DsrB, DsrC, DsrMKJOP, and QmoABC). Some of these enzymes may participate in the oxidative branch of the sulfur disproportionation pathway. Genes possibly involved in sulfur disproportionation—including the YTD gene cluster (*yedE*, *tusA*, and *dsrE*) (14), Eyh (15), and the MOLY cluster (16)—are encoded. The sulfur oxidation genes SoxA, SoxB, SoxX, SoxY, and SoxZ were also identified.

## Data Availability

This whole genome shotgun project has been deposited at DDBJ/ENA/GenBank under accession number JBMERE000000000.1. Fastq files of raw reads were deposited in NCBI SRA under accession numbers SRR32896280 and SRR32896281. Custom HMM profiles were deposited in Zenodo: https://doi.org/10.5281/zenodo.15383769.
